# Management of Choledocholithiasis After a Gastric Bypass Surgery: A Case Report

**DOI:** 10.7759/cureus.77337

**Published:** 2025-01-12

**Authors:** Mohamed A Ahmed, Rasha Saeed, Nathaniel Wolf, Stylianos Tsintzilonis, Danya Auda

**Affiliations:** 1 Surgery, AdventHealth Florida, Tampa, USA; 2 Occupational Medicine/Environmental Medicine, University of California, Irvine, USA; 3 Psychology, University of California, Riverside, USA

**Keywords:** abdominal sepsis, abnormal liver function test, choledocholithiasis, laparoscopic roux-en-y gastric bypass, percutaneous transhepatic cholangiogram

## Abstract

Alteration of the anatomy following Roux-en-Y gastric bypass (RYGB) poses a significant challenge in choledocholithiasis management in patients who have undergone this operation. Many options exist, including endoscopic, surgical, percutaneous, or hybrid means with variable success rates. Treatment should progress from least to most invasive options. We present a case of choledocholithiasis two years following gastric bypass managed with percutaneous transhepatic stone advancement to the duodenum. The aim is to highlight different options for dealing with this pathology.

## Introduction

Gallstones are common in patients following laparoscopic Roux-en-Y gastric bypass (LRYGB) with an incidence of 37% [1]. The pathophysiology of gallstone disease following bariatric surgery is due to a combination of large weight loss leading to biliary cholesterol saturation and alterations in biochemical triggers for gallbladder emptying, which promotes bile stasis [2]. While the incidence of cholelithiasis after bariatric surgery is quite high, the number of patients who develop symptomatic gallstone disease is variable. Choledocholithiasis is a rare complication after LRYGB with a 0.2% incidence; however, it represents an important therapeutic challenge due to the anatomical modifications of the gastrointestinal tract [3]. Therapeutic modalities for the management of choledocholithiasis following LRYGB include laparoscopy-assisted transgastric endoscopic retrograde cholangiopancreatography (ERCP), balloon enteroscopy-assisted ERCP, percutaneous biliary drainage with subsequent transfistula treatment, laparoscopic exploration of the common bile duct, endoscopic ultrasound (EUS)-guided intrahepatic puncture with antegrade clearance, percutaneous transhepatic biliary drainage, rendezvous guidewire-associated ERCP, and ultrasound-directed transgastric ERCP [4]. Each treatment modality presents its own set of advantages, technical challenges, logistical issues, and complications, all of which remain beyond the scope of this manuscript. 

## Case presentation

A 48-year-old male patient presented to our emergency room with diffuse abdominal pain following a fatty meal. His medical history included non-insulin-dependent diabetes, hypertension, sleep apnea, and obesity status post Roux-en-Y gastric bypass (RYGB) two years prior. On presentation, laboratory findings revealed elevated liver function tests (LFTs) (Table 1).

**Table 1:** The Patient's Liver Function Tests on Admission U/L: units per liter; dl: deciliter; mg: milligram

Liver Function Parameter	Measured Value	Reference Range
Aspartate Transferase (AST)	186 u/dL	13 - 40 u/dL
Alanine Aminotransferase (ALT)	88 u/dL	< 40 u/dL
Alkaline Phosphatase (ALP)	148 u/dL	44 - 147 u/dL
Total Bilirubin	2.6 mg/dL	0.3 - 1.2 u/dL

**Figure 1:**
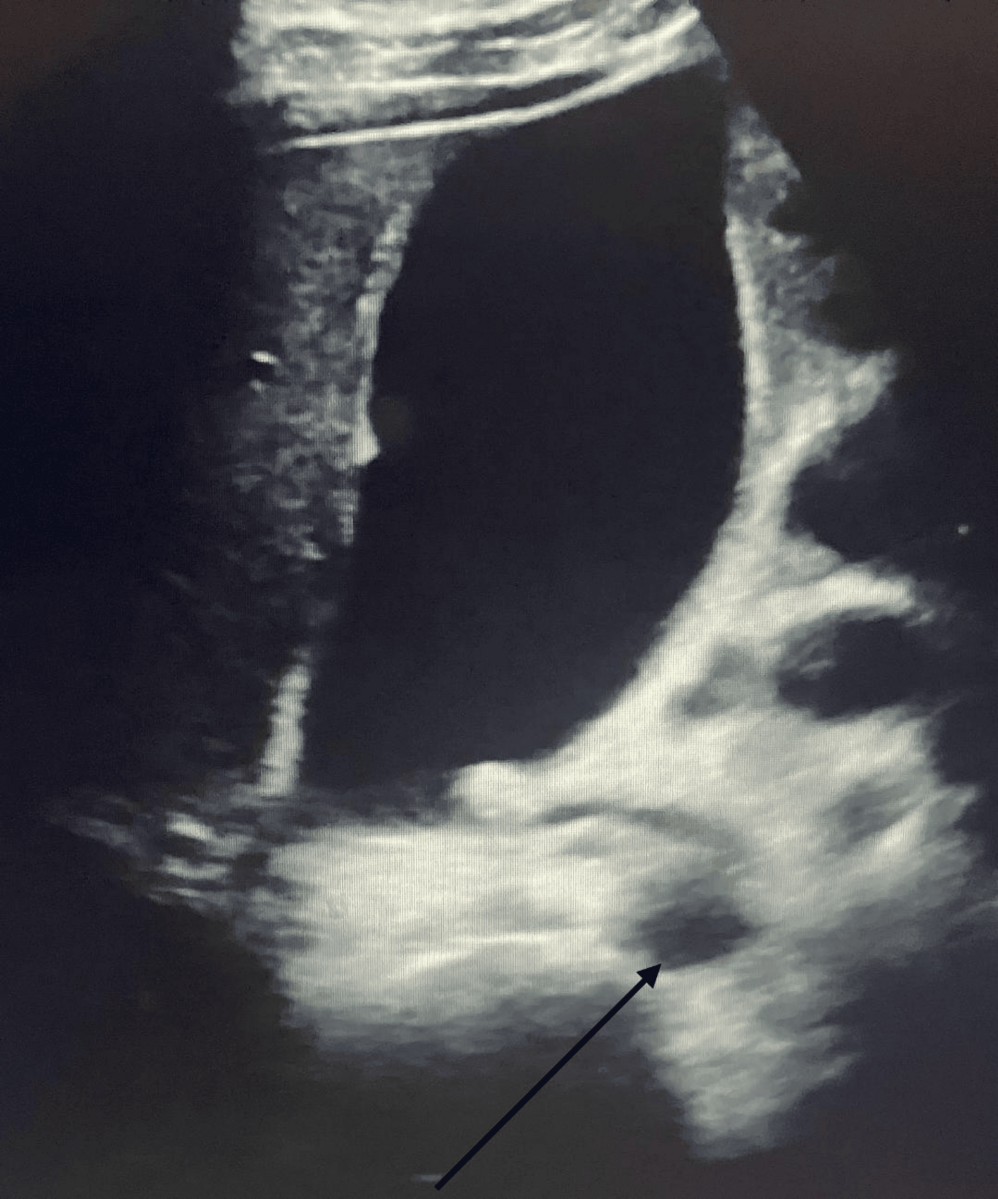
Ultrasound of the Abdomen The black arrow shows a dilated common bile duct measuring 5.8 mm. There are no stones in the gallbladder and no signs of acute cholecystitis.

Abdominal ultrasound evaluation revealed a prominent common bile duct dilated to 5.8 mm and no cholelithiasis or signs of acute cholecystitis (Figure 1)

**Figure 2:**
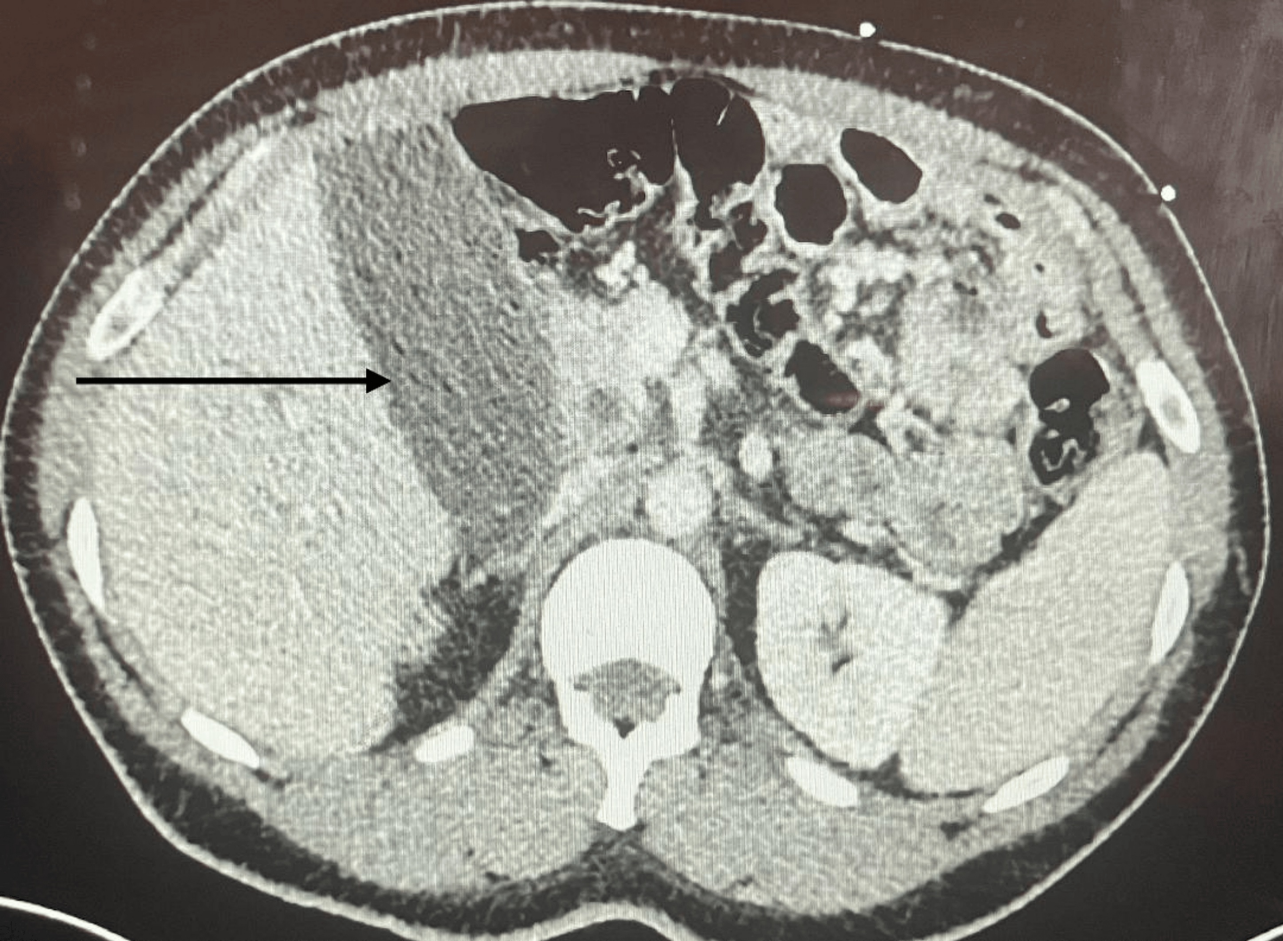
Computerized Tomography of the Abdomen The black arrow indicates a distended gallbladder with no acute findings.

Computerized tomography (CT) of the abdomen showed a moderately distended gallbladder, but no other acute findings (Figure 2).

The following day, he was noted to have rising LFTs (Table 2).

**Table 2:** Liver Function Tests: Hospital Day 1 U/L: units per liter; dl: deciliter; mg: milligram

Liver Function Parameter	Measured Value	Reference Range
Aspartate Transferase (AST)	380 u/dL	13 - 40 u/dL
Alanine Aminotransferase (ALT)	218 u/dL	< 40 u/dL
Alkaline Phosphatase (ALP)	143 u/dL	44 - 147 u/dL
Total Bilirubin	3.2 mg/dL	0.3 - 1.2 u/dL

**Figure 3:**
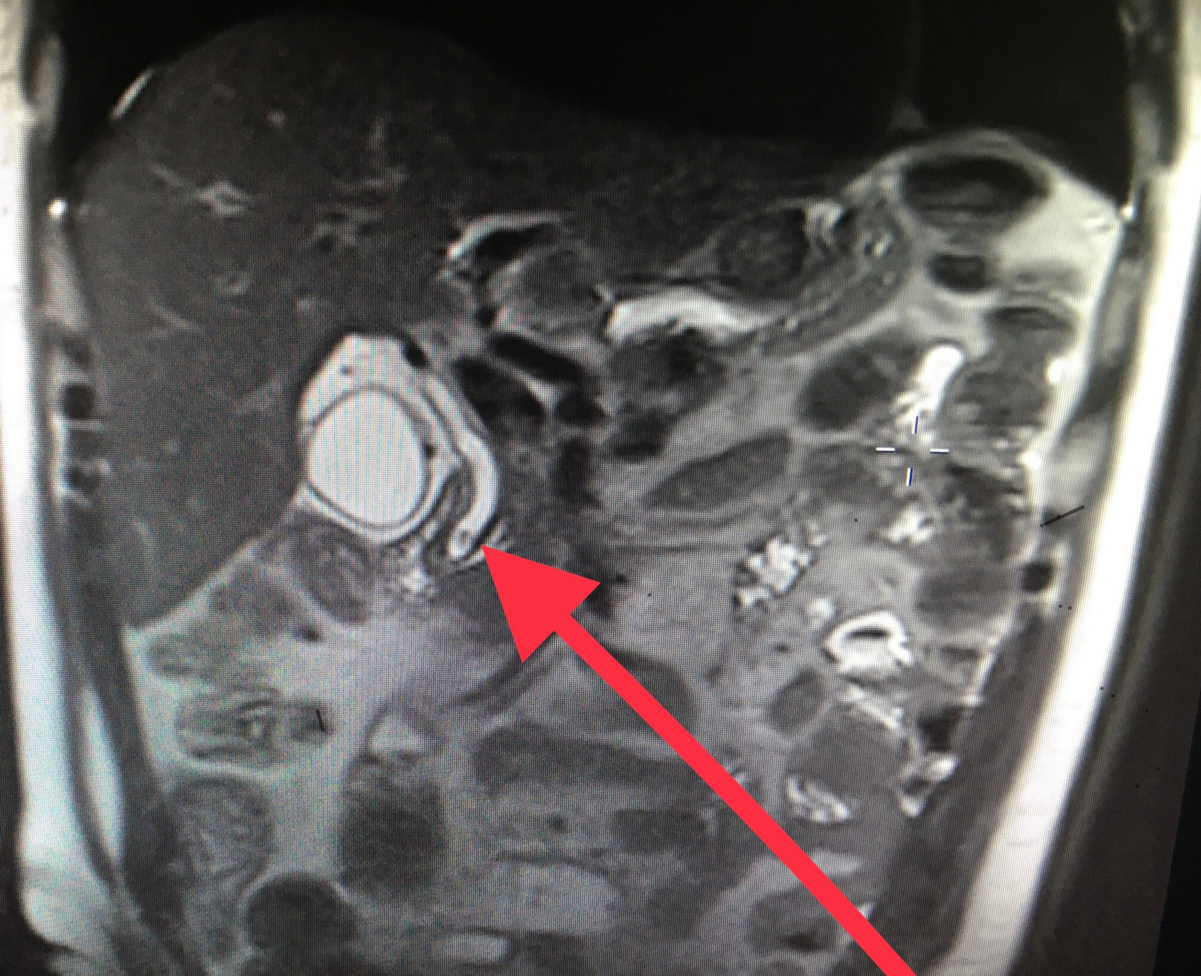
Magnetic Resonance Cholangiopancreatography (MRCP) Images The common bile duct stone is indicated by the red arrow.

Magnetic resonance cholangiography (MRCP) was performed to evaluate potential etiologies of the increasing LFTs despite the abdominal ultrasound and CT findings. The MRCP revealed cholelithiasis and choledocholithiasis (7 mm common bile duct stone) with mild intrahepatic and common bile duct dilatation and a distended gallbladder with wall thickening (Figure 3). 

**Figure 4:**
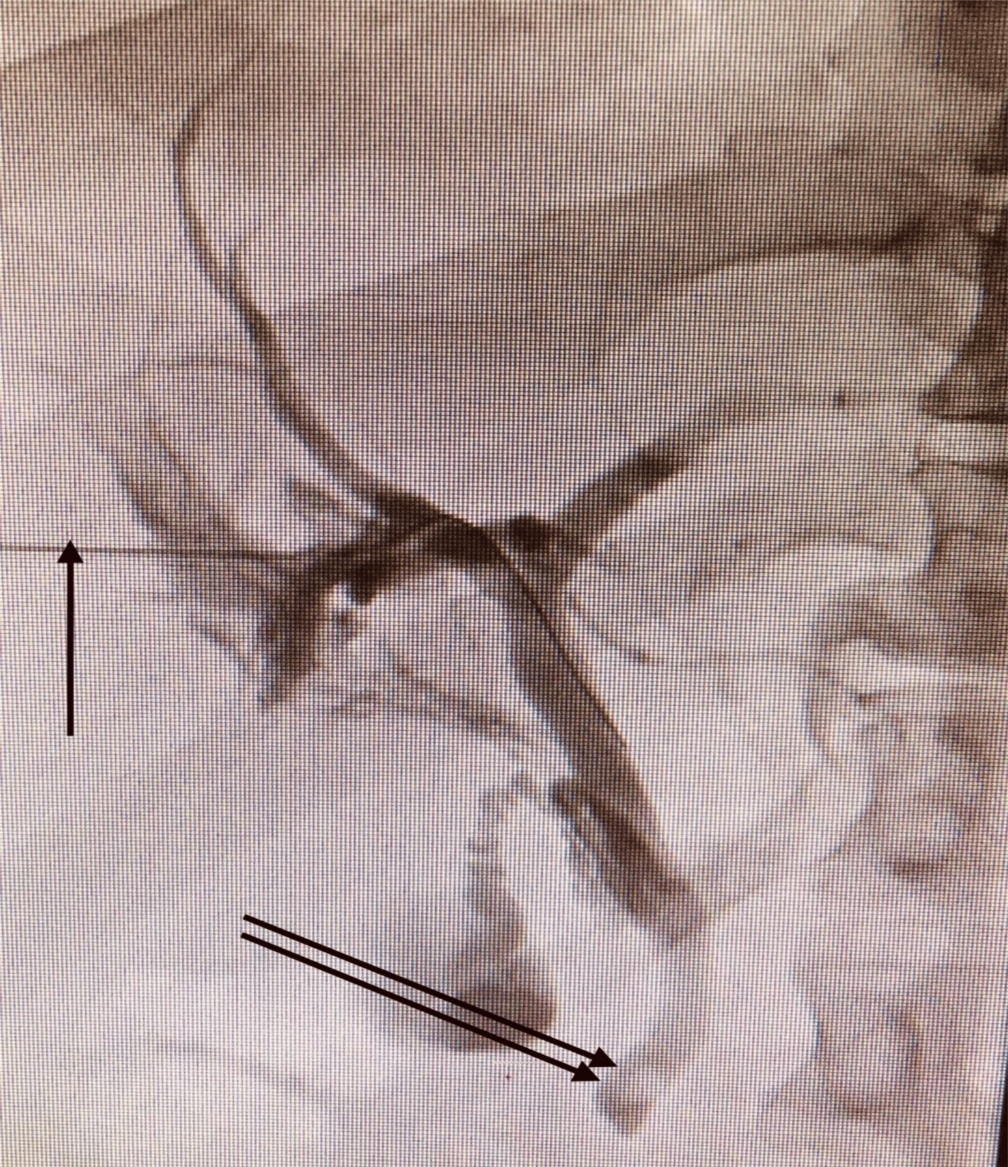
Percutaneous Transhepatic Cholangiography The percutaneous needle is indicated by one arrow); the common bile duct stone is indicated by two arrows.

**Figure 5:**
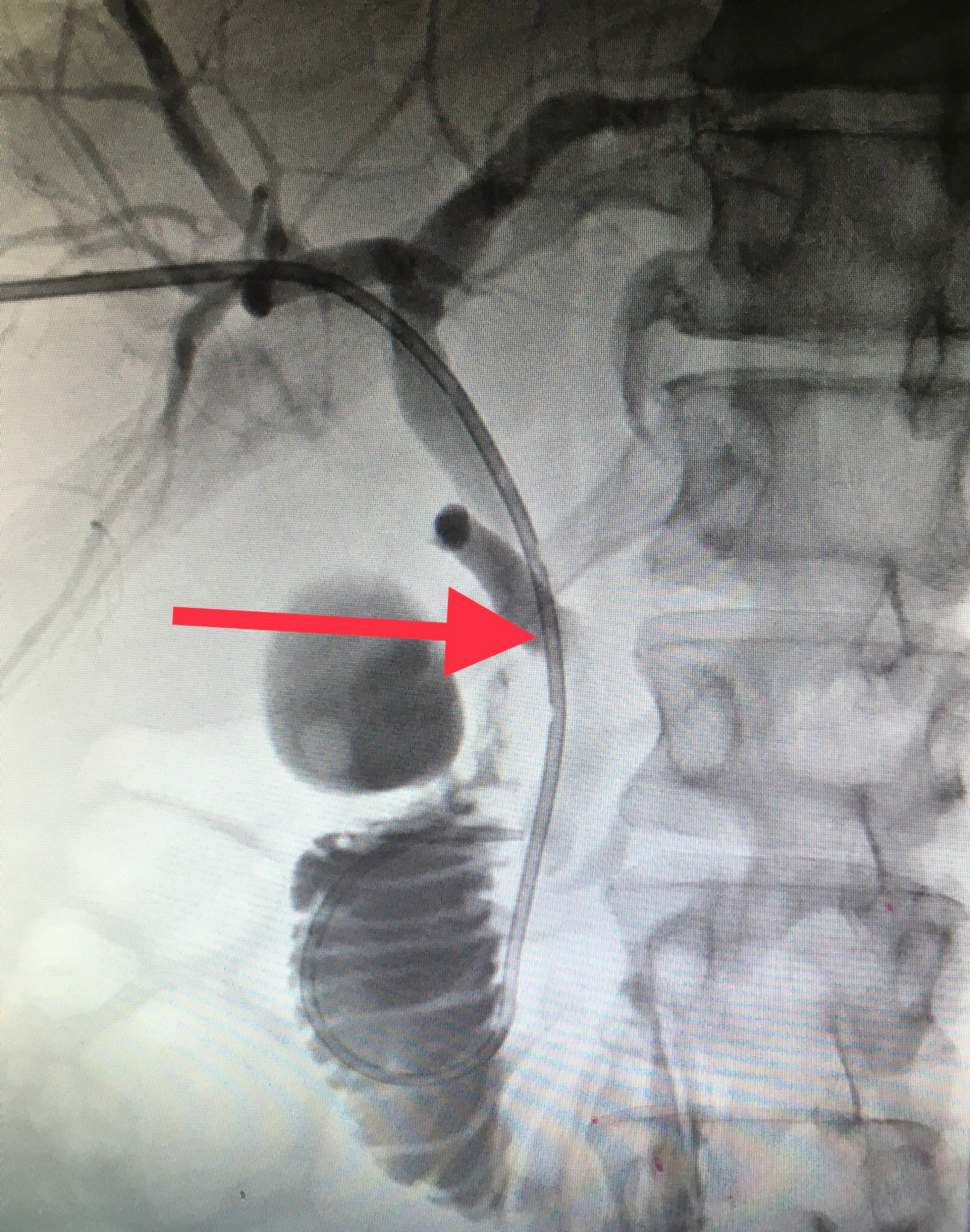
Percutaneous Common Bile Duct Stent Placement The common bile duct stone was advanced to the duodenum, and a percutaneous stent was placed as indicated by the red arrow.

Percutaneous cholangiography was performed with dislodgement of the common bile duct stone and stent (internal-external drain) placement (Figures 4, 5). 

The following day, LFTs normalized, and laparoscopic cholecystectomy was performed. The patient recovered well and was discharged from the hospital the following day with the planned removal of the transhepatic stent in four weeks.

## Discussion

As mentioned in the introductory section, the incidence of cholelithiasis in patients who have undergone bariatric surgery, particularly RYGB, raises the question of the utility of prophylactic cholecystectomy in this patient population at the time of their bariatric operation to avoid the issue of symptomatic disease entirely [5]. A meta-analysis by Warschkow provided guidance as it relates to cholecystectomy during LRYGB in obese patients, which was proven to be unjustified [6]. Another systematic review of this topic by Tutsumi et al. points out that while some surgeons justify concomitant cholecystectomy to avoid symptomatic gallstone disease, cholecystectomy in this situation poses a unique set of challenges and safety concerns. These challenges include suboptimal trocar placement, the difficulty of dissection of Calot’s triangle obscured with large amounts of visceral fat, and the large size of the often-present steatotic liver obscuring visualization [7]. In our case, we faced the dilemma of dealing with biliary obstruction after a gastric bypass procedure.

Biliary tract obstruction secondary to a stone, while causing pain primarily, can also result in acute cholangitis (infection in the biliary tract) with a wide variety of bacteria, mainly *Escherichia coli* and *Klebsiella*. Despite improved management, mortality rates due to acute cholangitis remain at approximately 5% [8]. Antimicrobial therapy and endoscopic or radiological biliary drainage are crucial management components. Anatomical alteration following gastric bypass presents a biliary drainage challenge and can dictate the preferred procedure. In patients with Roux and biliopancreatic limbs less than 150 cm, deep enteroscopy-assisted ERCP should be the first-line procedure, while laparoscopic-assisted ERCP should be offered in cases where the limb is equal to or greater than 150 cm [9]. Laparoscopic common bile duct exploration is another option when expertise is available with an 84% success rate and an 8% conversion to open rate [10].

Endoscopic ultrasound-guided intrahepatic puncture with antegrade clearance after the creation of the hepaticoenteric tract by EUS-guided hepaticogastrostomy followed by mechanical lithotripsy a month later once the newly created tract matures has also been described [11]. Rendezvous guidewire-associated ERCP has a higher success rate than balloon-assisted enteroscopy alone and requires the passage of a percutaneous transhepatic guidewire, which will direct balloon enteroscopy [12]. Percutaneous transhepatic cholangioscopy with intraductal electrohydraulic lithotripsy for the management of choledocholithiasis in an inaccessible papilla has been described [13]. The percutaneous transhepatic technique requires a local anesthetic, and minimal intravenous sedation can be effective, less costly, and with very good success rates when experts are available [14]. 

## Conclusions

Choledocholithiasis following gastric bypass present a management dilemma. Different options have been described with varying degrees of success and institutional skills availability. Treatment should progress from least to most invasive options. Percutaneous transhepatic choledochography with stone dislodgement and common bile duct stenting appears to be a very viable option when feasible.
